# Echocardiographic Epicardial Adipose Tissue Thickness for Risk Stratification of Patients With Heart Failure

**DOI:** 10.3389/fphys.2020.00043

**Published:** 2020-02-05

**Authors:** Valentina Parisi, Maddalena Conte, Laura Petraglia, Fabrizio Vincenzo Grieco, Dario Bruzzese, Aurelio Caruso, Maria Gabriella Grimaldi, Pasquale Campana, Paola Gargiulo, Stefania Paolillo, Emilio Attena, Vincenzo Russo, Gennaro Galasso, Antonio Rapacciuolo, Pasquale Perrone Filardi, Dario Leosco

**Affiliations:** ^1^Department of Translational Medical Sciences, University of Naples Federico II, Naples, Italy; ^2^Department of Public Health, University of Naples Federico II, Naples, Italy; ^3^Department of Cardiology and Cardiac Surgery, Casa di Cura San Michele, Maddaloni, Italy; ^4^Department of Advanced Biomedical Sciences, University of Naples Federico II, Naples, Italy; ^5^Department of Cardiology, San Giuliano Hospital, Giugliano in Campania, Italy; ^6^Department of Translational Medical Sciences, University of Campania “Luigi Vanvitelli”, Monaldi Hospital, Naples, Italy; ^7^Department of Cardiology, San Giovanni di Dio e Ruggi d’Aragona Hospital, Salerno, Italy

**Keywords:** epicardial adipose tissue, heart failure prognosis, echocardiography, implantable cardioverter defibrillator, arrhythmias

## Abstract

**Background and Aims:**

Epicardial adipose tissue (EAT) has been shown to be involved in the pathogenesis and progression of heart failure (HF). In this study we aimed to explore the predictive value of echocardiographic EAT thickness on prognosis of a selected population of HF patients.

**Methods:**

The patient population included n. 69 consecutive patients with systolic HF referred to implantable cardioverter defibrillator (ICD) implantation for primary or secondary prevention. At the time of enrolment, echocardiographic EAT thickness was assessed in all patients along with demographic and clinical data. The study had a median follow-up time of 49.8 months. We assessed the prognostic predictive value of EAT thickness on a composite clinical and arrhythmic outcome including HF related deaths, new hospital admissions for HF worsening, and atrial and life threatening ventricular arrhythmic events. Clinical and arrhythmic outcomes were also evaluated separately.

**Results:**

At univariate analysis, EAT thickness significantly predicted all the three outcomes considered. Of interest, at multivariate analysis, after adjusting for known risk factor, EAT remained significantly associated to the composite [HR 1.18 (1.09–1.28); *p* < 0.001], arrhythmic [HR 1.14 (1.03–1.25); *p* = 0.008], and clinical [HR 1.14 (1.03–1.27); *p* = 0.010] outcomes.

**Conclusion:**

Echocardiographic assessment of EAT can predict outcome of HF patients and it is significantly associated with both arrhythmic and clinical events. These preliminary findings pave the way for future and larger studies aimed to definitively recognize the prognostic value of this novel risk marker in HF.

## Introduction

Echocardiography represents a gold standard for the assessment of patients with systolic heart failure (HF) and most of clinical and therapeutic decisions are based on the values of left ventricular ejection fraction (LVEF) obtained through this technique. The current guidelines indicate a LVEF ≤ 35% as the cut off value to identify patients at higher risk of HF progression and sudden death (Ponikowski et al., 2016). However, less than one third of implantable cardioverter defibrillator (ICD) recipients in primary prevention receives appropriate therapy, thus suggesting as a reduced LVEF could not always be associated to an arrhythmogenic substrate (Van der Bijl et al., 2017). Furthermore, although LVEF undoubtedly represents an important prognostic risk marker, in many cases it cannot express all the complex metabolic and neurohormonal alterations associated with HF prognosis. Recent evidence highlight the role of epicardial adipose tissue (EAT) in HF pathogenesis and progression (Parisi et al., 2016; Packer, 2018; Ansaldo et al., 2019). We have previously reported that EAT is increased in patients with systolic HF and may concur to HF-related cardiac adrenergic derangement being a local source of catecholamines (Parisi et al., 2016). Furthermore, an EAT pro-inflammatory phenotype can affect the electrophysiological properties of myocytes and induce fibrosis, thus promoting arrhythmogenesis and HF progression (Iacobellis et al., 2005; Lin et al., 2012).

Although several evidence support the pathophysiological implication of EAT in HF, the prognostic value of EAT accumulation in HF patients is still unknown. To this aim, in the present study, we explored the predictive value of echocardiographic EAT thickness on clinical and arrhythmic outcomes of a high-risk HF population.

## Materials and Methods

### Study Population

The study population included 69 consecutive patients with systolic HF referred to ICD implantation for primary or secondary prevention, recruited at the HF clinic of Federico II University of Naples, in accordance to the current HF guidelines of the European Society of Cardiology (Ponikowski et al., 2016). The inclusion criteria were: (i) patients enrolled for ICD implantation in primary prevention, defined as symptomatic HF patients (NYHA Class II–III) with a LVEF ≤35% despite ≥3 months of optimal medical therapy, and without acute coronary syndromes in the last 3 months; (ii) patients enrolled for ICD implantation in secondary prevention, defined as patients who have recovered from a ventricular arrhythmia causing haemodynamic instability, and who were expected to survive more than one year with good functional status. We excluded patients with conditions known to interfere with systemic and local EAT inflammation and associated to EAT thickness increase such as moderate to severe valvular disease, chronic atrial fibrillation, myocardial inflammatory diseases, cancer and or systemic inflammatory diseases.

At the time of enrolment and before ICD implantation, all patients underwent a complete clinical examination. Demographic data, including age, sex, HF medications, cardiovascular risk factors and the presence of comorbidities were also collected.

The study was approved by the local Ethics Committee. All procedures performed in the study were in accordance with the ethical standards of the institutional or national research committee and with the 1964 Helsinki declaration and its later amendments or comparable ethical standards and conformed to the Declaration of Helsinki on human research. All patients included in the study gave written informed consent after receiving an accurate explanation of the study protocol and of the potential risks related to the procedures adopted by the study.

### Echocardiographic Study

**FIGURE 1:**
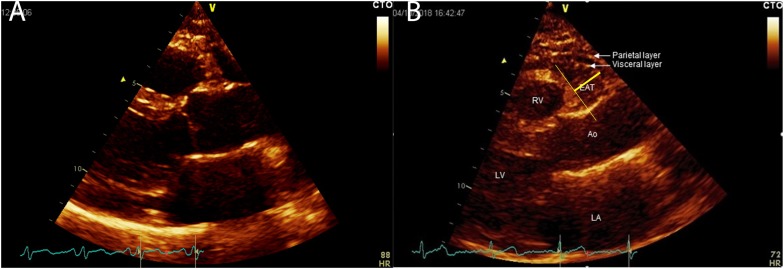
EAT thickness visualization and measurement in parasternal long axis view; panel **(A)** thin epicardial adipose tissue; panel **(B)** thick epicardial adipose tissue. EAT, Epicardial Adipose Tissue; LA, left atrium; LV, left ventricle; Ao, ascending aorta; RV, right ventricle. EAT is visualized between the visceral layer of the pericardium an the myocardium at the Rindfleisch fold.

All patients underwent a complete echocardiographic examination including EAT measurement. Echocardiograms were performed by a VIVID E9 (GE Healthcare) machine, according to standard techniques. EAT thickness was obtained, as previously described (Parisi et al., 2019b). EAT was visualized in parasternal long-axis view between the free wall of the right ventricle and the anterior surface of the ascending aorta. To improve image quality, the depth was reduced, the focus adjusted, and ultrasound beam frequency slightly increased. The colorimetric map was switched into gold. Once visualized the EAT deposit, the maximum EAT thickness was measured at end-systole (Figure 1). The average value from 3 cardiac cycles was used for the statistical analysis.

### Follow Up and Outcomes

All patients underwent a 6-month follow-up. At any follow-up visit, patients underwent clinical examination and ICD check. We considered a composite clinical and arrhythmic outcome including HF related deaths, new hospital admissions for HF worsening, and atrial and ventricular arrhythmic events (atrial tachycardia and/or atrial fibrillation lasting more than 30 s, ventricular tachycardia lasting more than 8 beats and episodes of ventricular fibrillation, shock-treated or self-terminating). Clinical and arrhythmic outcomes were also evaluated separately.

### Statistical Analysis

Numerical variables were described using mean ± standard deviation with range while categorical factors were synthetized using absolute frequencies and percentages. Baseline correlations among clinical and demographical characteristics were estimated using the Pearson correlation coefficient. Events free survival was defined as the time between and the first occurrence of any of the cardiovascular events assessed in the present study (arrhythmic, clinical or both) or censoring, whichever occurred first. Incidence rates were estimated using the cumulative time at risk at denominator and the number of patients with at least one event as numerator. The corresponding 95% confidence intervals (95% C.I) were computed assuming a Poisson distribution. The association between EAT, LVEF and the risk of cardiovascular events was assessed using univariate and multivariable Cox regression while the cumulative probability of events was estimated, and graphically represented, using the Kaplan–Meier method. Statistical significance was accepted at *p* < 0.05.

## Results

Table 1 illustrates demographic, clinical, and echocardiographic data of the whole population. The mean age was 64.5 ± 10.4 (range 40–90) years and 88.4% of patients were men.

**TABLE 1:** Demographic and clinical data of the study population.

	**Overall cohort (n. 69 pts)**
Age, yrs	64.5 ± 10 (40–90)
Gender, Male, n (%)	61 (88.4)
NYHA class (%)	–
I	2 (2.9)
II	46 (66.7)
III	21 (30.4)
**Cardiovascular risk factors**	
Smokers, n (%)	43 (63.2)
Diabetes, n (%)	36 (52.2)
Hypertension, n (%)	47 (68.1)
Dyslipidemia, n (%)	48 (69.6)
Ischemic cause of HF, n (%)	47 (68.1)
Echocardiographic EAT thickness (mm) mean ± SD	10.2 ± 3.6 (4 to 20)
LVEF (%), mean ± SD	35.7 ± 7.8 (20 to 64)
LVEF ≤ 35%, n (%)	36 (52.9)
**HF drug therapy**	
Beta blockers, n (%)	52 (75.4)
ACE I/ARB, n (%)	54 (78.6)
Aldosterone antagonists	31 (44.9)

*NYHA, New York heart association; HF, heart failure; EAT, epicardial adipose tissue; LVEF, left ventricular ejection fraction; ACEi, angiotensin converting enzyme inhibitors; ARB, angiotensin receptor blockers.*

Most of patients were in NYHA class II (66.7%) and III (30.4%). Ischemic cause of HF was recognized in 47.1% of cases. Fifty-two percent of patients were diabetics, 68.1% had hypertension, 63.2% were smokers, and 69.6% had dyslipidaemia. Most of patients were on optimal HF drug therapy. Mean LVEF was 35.7 ± 7.8%, and was ≤ 35% in 52.9% of patients. Mean EAT thickness was 10.2 ± 3.6 (range: 4–20) mm.

Epicardial adipose tissue thickness did not correlate with LVEF (*r* = −0.11, *p* = 0.369), whereas it showed a significant correlation with age (*r* = 0.25, *p* = 0.037) and left atrial volume (*r* = 0.29. *p* = 0.027). EAT thickness was not associated with diabetes (*p* = 0.278), hypertension (*p* = 0.521), dyslipidemia (*p* = 0.532) and smoking habit (*p* = 0.139). No correlation was found between EAT thickness and BMI (*r* = 0.02, *p* = 0.868).

### Number and Type of Events

After ICD implantation, all patients underwent 6-month follow up visits with a median follow-up period of 49.8 months [range: 3.1–74.4 months]. There were n. 30 HF related hospitalizations, and 5 HF related deaths. ICD check allowed to recognize n. 7 episodes of sustained atrial tachycardia, n. 20 episodes of atrial fibrillation lasting more than 30 s, n. 14 episodes of sustained ventricular tachycardia and 5 episodes of ventricular fibrillation. All arrhythmic ventricular events were succesfully treated by antitachycardia pacing and/or ICD shock therapy.

### Predictors of Outcome

At univariate analysis, we tested the predictive value of the following variables: age, sex, BMI, diabetes, hypertension, smoking, NYHA class, LVEF, echocardiographic EAT thickness, HF ischaemic etiology, use of angiotensin-converting enzyme inhibitors or sartans and β-blockers. Using parsimonious criteria and taking into account the study sample size, 9 potentially prognostic independent variables have been selected for the multivariate analysis. The independent role of echocardiographic EAT thickness was assessed after adjusting each model for age, gender, BMI, diabetes, hypertension, smoking, LVEF and use of β-blockers.

#### Composite Outcome

At univariate analysis, EAT was significantly associated with the outcome, showing, for each unit increase, a 16% increment in the hazard of the composite event [H.R. 1.16; 95% C.I (1.08–1.24); *p* < 0.001]. A LVEF value below 35% determined about a two-fold increase in the hazard of the composite event [H.R. 1.91; 95% C.I (1.08–3.37), *p* = 0.026]. Of interest, at multivariate analysis, after adjusting for known risk factor, EAT remained significantly associated to the composite outcome while LVEF did not. When the analysis was repeated excluding atrial events, the results were almost the same; LVEF and EAT were both predictors of outcome at univariate analysis (*p* = 0.003 and *p* = 0.026 respectively), while only EAT remained a significant predictor at multivariate analysis (*p* = 0.008). BMI, diabetes and β-blockers were predictors of the composite outcome at the multivariate analysis (Table 2).

**TABLE 2:** Predictors of the composite outcome.

	**Composite outcome**			
	**Univariate**		**Multivariable**	
	**HR (95% C.I)**	***p***	**HR (95% C.I)**	***p***
Age (years)	1.01(0.98−1.04)	0.417	0.98(0.95−1.02)	0.386
Gender; male	0.56(0.25−1.25)	0.156	0.46(0.18−1.23)	0.123
NYHA class; III	1.16(0.65−2.08)	0.614	−	−
BMI (kg/m2)	0.93(0.87−0.99)	0.033	0.88(0.81−0.95)	0.002
Diabetes	1.41(0.81−2.45)	0.221	2.01(1.05−3.82)	0.034
Hypertension	0.94(0.53−1.67)	0.833	1.16(0.61−2.23)	0.651
Smokers	1.6(0.6−1.87)	0.84	1.23(0.6−2.54)	0.572
Ischemic cause of HF	1.01(0.56−1.82)	0.976	−	−
EAT thickness (mm)	1.16(1.08−1.24)	< 0.001	1.18(1.09−1.27)	< 0.001
LVEF ≤ 35%	1.91(1.08−3.37)	0.026	1.59(0.82−3.08)	0.168
Beta blockers	0.46(0.25−0.84)	0.012	0.46(0.23−0.91)	0.025
ACE I/ARB	1.09(0.63−1.89)	0.763	−	−

*NYHA, New York heart association; BMI, body mass index; HF, heart failure; EAT, epicardial adipose tissue; LVEF, left ventricular ejection fraction; ACEi, angiotensin converting enzyme inhibitors; ARB, angiotensin receptor blockers.*

Heart failure related hospitalizations represented the outcome with the highest number of events. To rule out the possibility that hospitalization was the only trigger for the significance observed in the composite outcome, we excluded from this outcome the hospitalization and, again, the significant and independent role of EAT emerged [HR: 1.12, 95% C.I (1.02–1.23), *p* = 0.021].

#### Arrhythmic Events

At univariate analysis, only EAT was associated with an increased risk of arrhythmic events with an HR equa1 to 1.12, [95% C.I (1.03–1.22); *p* = 0.008]. Even after adjusting for known risk factors, EAT remained a significant predictor with a 14% increase, for each unit increase, in the hazard of an arrhythmic event [HR: 1.14; 95% C.I (1.03–1.25), *p* = 0.008] (Table 3).

**TABLE 3:** Predictors of the arrhythmic outcome.

	**Arrhythmic outcome**			
	**Univariate**			
	**HR (95% C.I)**	***P*value**	**HR (95% C.I)**	***P*value**
Age (years)	1.02(0.98−1.06)	0.288	1(0.96−1.05)	0.891
Gender; male	0.6(0.23−1.57)	0.301	0.86(0.26−2.82)	0.805
NYHA class; III	1.34(0.66−2.72)	0.412	−	−
BMI (kg/m2)	092(0.85−1)	0.059	0.89(0.8−0.99)	0.025
Diabetes	1.15(0.58−2.27)	0.692	1.61(0.73−3.57)	0.241
Hypertension	0.74(0.37−1.49)	0.401	0.81(0.38−1.76)	0.599
Smokers	0.94(0.47−1.87)	0.853	1.19(0.5−2.82)	0.694
Ischemic cause of HF	0.83(0.41−1.67)	0.595	−	−
EAT thickness (mm)	1.12(1.03−1.22)	0.008	1.14(1.03−1.25)	0.011
LVEF ≤ 35%	1.31(0.66−2.61)	0.445	0.96(0.43−2.13)	0.921
Beta blockers	0.44(0.21−0.9)	0.025	0.47(0.21−1.02)	0.055
ACE I/ARB	1.29(0.64−2.58)	0.476	−	−

*NYHA, New York heart association; BMI, body mass index; HF, heart failure; EAT, epicardial adipose tissue; LVEF, left ventricular ejection fraction; ACEi, angiotensin converting enzyme inhibitors; ARB, angiotensin receptor blockers.*

#### Clinical Events

At univariate analysis, LVEF and EAT were both predictors of events [HR: 2.43, 95% C.I (1.16–5.09), *p* = 0.018 and HR: 1.13, 95% C.I (1.03–1.23), *p* = 0.010; respectively]. When both variables were considered in the same model adjusted for known risk factors, EAT remained the only significant predictor [HR: 1.14, 95% C.I (1.03–1.27), *p* = 0.010] (Table 4). A sensitivity analysis was performed to assess separately the predictive value of EAT on HF hospitalizations, the outcome with most frequent events. We found a significant and independent prognostic role of EAT, with a 13% increase in the hazard of hospitalization for every unit increase of EAT [HR: 1.13, 95% C.I (1.01–1.26), *p* = 0.034].

**TABLE 4:** Predictors of the clinical outcome.

	**Clinical outcome**			
	**Univariate**			
	**HR (95% C.I)**	***p*value**	**HR (95% C.I)**	***p*value**
Age (years)	0.99(0.96−1.03)	0.722	0.97(0.93−1.02)	0.201
Gender; male	0.84(0.29−2.43)	0.754	0.43(0.11−1.71)	0.229
NYHA class; III	1.07(0.51−2.26)	0.861	−	−
BMI (kg/m2)	0.94(0.86−1.02)	0.148	0.9(0.79−1.02)	0.105
Diabetes	1.81(0.89−3.68)	0.104	2.2(0.97−4.97)	0.059
Hypertension	1.67(0.75−3.73)	0.209	2.56(1−6.53)	0.05
Smokers	1.14(0.55−2.35)	0.727	1.11(0.44−2.82)	0.827
Ischemic cause of HF	1.56(0.67−3.61)	0.299	−	−
EAT thickness (mm)	1.13(1.03−1.23)	0.01	1.14(1.03−1.27)	0.011
LVEF ≤ 35%	2.43(1.16−5.09)	0.018	2.28(1−5.22)	0.051
Beta blockers	0.95(0.41−2.2)	0.898	1.06(0.42−2.71)	0.896
ACE I/ARB	0.79(0.39−1.57)	0.495	−	−

*NYHA, New York Heart Association; BMI, body mass index; HF, heart failure; EAT, epicardial adipose tissue; LVEF, left ventricular ejection fraction; ACEi, angiotensin converting enzyme inhibitors; ARB, angiotensin receptor blockers.*

**FIGURE 2:**
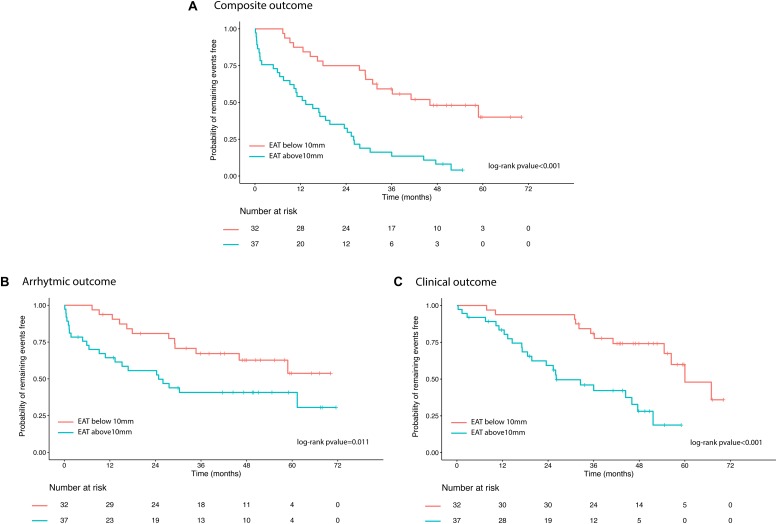
Kaplan Meiers curves illustrating the probability of remaining events free for each of the three considered outcomes [panel **(A)** composite outcome; panel **(B)** arrhythmic outcome; panel **(C)** clinical outcome] and stratified according to EAT median value. EAT, epicardial adipose tissue.

Kaplan Meiers estimates of the probability of remaining events free for each of the three considered outcomes and stratified according to EAT median value are shown in Figure 2.

## Discussion

The main finding of the present study is that echocardiographic measurement of EAT predicts outcome in high risk HF patients. In particular, EAT is associated with both arrhythmic and clinical events.

Epicardial adipose tissue represents the visceral fat depot of the heart which covers 80% of the heart’s surface (Iacobellis et al., 2005). In pathologic conditions, EAT may play an unfavorable activity for the heart through production and secretion of proinflammatory and proatherogenic mediators (Baker et al., 2006) and is associated with atherosclerotic diseases, such as coronary artery disease, and aortic stenosis (Chaldakov et al., 2001; Eroglu et al., 2009; Parisi et al., 2015, 2019a; Liguori et al., 2018). Local levels of EAT-secreted inflammatory mediators are higher than those observed in the subcutaneous adipose tissue and are independent from the presence of obesity, and diabetes (Mazurek et al., 2003). EAT obtained from patients with coronary artery disease shows a greater inflammatory infiltrate compared to subcutaneous adipose tissue and activation of both innate and adaptive immunity (Mazurek et al., 2003; Baker, 2009). Moreover, human EAT shows higher mRNA expression of proteins involved in oxidative stress (Salgado-Somoza et al., 2010). Oxidative stress and the specific regulation of signaling pathways by reactive oxygen species (ROS) are increasingly recognized as important contributors to the pathophysiology of HF, influencing many key aspects of the failing heart phenotype such as hypertrophy, matrix remodeling, contractile dysfunction, endothelial dysfunction, and arrhythmias (Hafstad et al., 2013). There is a growing body of evidence that immune activation and inflammation play a crucial role in the progression of LV dysfunction (Heymans et al., 2009; Wagner et al., 2014). Thus, it seems to be reasonable to hypothesize that EAT, through its potent pro-atherogenic, pro-inflammatory, pro-oxidant activities, may contribute to HF pathogenesis and influence the progression of LV dysfunction in HF patients.

Epicardial adipose tissue is associated with LV hypertrophy and diastolic dysfunction (Iacobellis et al., 2004; Fontes-Carvalho et al., 2014). Importantly, it has been demonstrated that human EAT can induce myocardial fibrosis through secretion of adipofibrokines, such as Activin A (Venteclef et al., 2015). In this regard, it is known that myocardial fibrosis represents an important pathophysiological mechanism involved in HF development and progression (Schelbert et al., 2015; González et al., 2018) and it may be a trigger for both atrial and ventricular arrhythmias (Gulati et al., 2013; Perazzolo Marra et al., 2014). Therefore, EAT could contribute to the formation of an unfavorable pro-arrhythmic substrate conditioning HF adverse prognosis. The pro-arrhythmic activity of EAT could be also explained by its contribution to enhance the adrenergic nervous system derangement in HF. We have previously demonstrated that EAT is a local source of catecholamines and its echocardiographic thickness is associated to myocardial adrenergic denervation at ^123*I–*^metaiodobenzylguanidine scintigraphy (Parisi et al., 2016), one of the most powerful imaging technique to assess HF prognosis (Jacobson et al., 2010). Furthermore, it has been demonstrated that EAT contains both adrenergic and cholinergic nerves which interact with the extrinsic nervous system (Ardell, 2011; Chen and Turker, 2012). Simultaneous activation of these nerve structures within EAT in response to extrinsic nerve activation may enhance triggered activity and facilitate the development of cardiac arrhythmias (Ferrara et al., 2005; Zhou et al., 2014; Jiang et al., 2015). Overall, these findings strongly support the EAT involvement in the pathophysiological processes affecting HF prognosis.

In the present study, we have reported, for the first time, that the simple echocardiographic measurement of EAT may be helpful to predict HF prognosis. We measured EAT at the Rindfleisch fold, a pericardial recess between the right ventricle and the aorta, where EAT measurement shows excellent reproducibility and well correlates with cardiac magnetic resonance EAT thickness and volume (Parisi et al., 2019b). At this level, the downward curvature of the right ventricle increases the space between the two pericardial layers, thus allowing the EAT expansion, its visualization and accurate measurement.

The population of this study was composed of HF patients enrolled at the time of ICD implantation for primary and secondary prevention. In this population, EAT thickness was independently associated with both clinical and arrhythmic outcomes. Importantly, EAT showed, for each unitary increase, a 16% increment in the hazard of the composite outcome and a 14% increment in the hazard of the arrhythmic outcome, thus confirming a powerful prognostic value in HF population. Regarding the unexpected lack of significance of LVEF on the different outcomes at multivariate analysis, we can only speculate that it might be ascribed to two distinct factors: (1) the capture of LVEF effect by the higher prognostic impact of EAT in the model; (2) the wide distribution of LVEF in the study population due to the inclusion of patients referred to ICD implantation in secondary prevention, thus showing, in many cases, a LVEF higher than 35%.

### Study Limitations

The major study limitation of the present study is represented by the relatively small sample size. However, we are confident that the results should not have been biased because of the small sample size. In fact, an insufficient sample size should move the results toward the null (i.e., no significant effect) rather than toward the alternative hypothesis. We are, at the same time, aware of the risk of inflating the probability of type I error due to the number of statistical models we used but, due to the consistency of the results for the different outcomes, we believe in their plausibility.

Our study population included patients with different HF etiologies (ischemic and non-ischemic) and with a skewed distribution of LVEF values. This might explain the lack of a significant prognostic impact of LVEF on the different outcomes considered.

The small sample size and the risk of increasing the probability of type-2 error, did not allowed us to separately analyze each of the outcomes considered. Notewhorthy, the number of deaths was very low, probably due to the selection of the study population (no patient died for sudden cardiac death given the ICD shock), thus avoiding to consider cardiac deaths as a separate outcome.

The main finding of the study is that EAT thickness is an independent predictor of outcome at multivariate analysis. This finding is highly novel and proposes EAT for HF risk stratification. However, larger studies are required to confirm our preliminary data and to assess the superiority of EAT thickness over other established markers of HF prognosis.

## Conclusion

The results of the present study suggest a potential role of echocardiographic EAT assessment in predicting clinical and arrhythmic outcomes in HF patients referred to ICD implantation for primary and secondary prevention. The pathophysiologic explanation of the association of EAT accumulation with HF prognosis relies in the pathologic properties of EAT able to promote cardiac fibrosis and adrenergic derangement that represent two important determinants of HF progression and contribute to the development of a pro-arrhythmic substrate.

To date, the selection of candidates to ICD implantation in primary prevention is still based on echocardiographic LVEF estimation. Nevertheless, only one third of ICD recipients with LVEF ≤35% receives appropriate therapy (Cacciatore et al., 2012; Takano et al., 2018), thus, we dramatically needed of novel markers, with higher sensitivity and specificity, for a better recognition of those HF patients who will really benefit from ICD therapy.

Given the recognized correlation of EAT with cardiac adrenergic denervation, a powerful indicator of worse HF prognosis, it seems to be reasonable to hypothesize that the echocardiographic assessment of cardiac visceral fat could represent, in the next future, a promising diagnostic tool to improve the HF risk stratification and the appropriateness of HF therapies.

## Data Availability Statement

The datasets generated for this study are available on request to the corresponding author.

## Ethics Statement

The studies involving human participants were reviewed and approved by Ethics Committee of the Federico II University of Naples. The patients/participants provided their written informed consent to participate in this study.

## Author Contributions

VP, MC, PP, and DL contributed to the conception and design of the study. LP, FG, DB, AC, MG, PC, EA, VR, and GG organized to the database. DB performed the statistical analysis. VP, SP, and AR wrote the first draft of the manuscript. VP, MC, PP, and DL wrote sections of the manuscript. All authors contributed to the manuscript revision, read and approved the submitted version of the manuscript.

## Conflict of Interest

The authors declare that the research was conducted in the absence of any commercial or financial relationships that could be construed as a potential conflict of interest.

## References

[B1] Ansaldo A. M. Montecucco F. Sahebkar A. Dallegri F. Carbone F. (2019). Epicardial adipose tissue and cardiovascular diseases. *Int. J. Cardiol.* 278 254–260. 10.1016/j.ijcard.2018.09.089 30297191

[B2] Ardell J. L. (2011). The cardiac neuronal hierarchy and susceptibility to arrhythmias. *Heart Rhythm.* 8 590–591. 10.1016/j.hrthm.2010.12.019 21167959 PMC3629697

[B3] Baker A. R. (2009). EAT as a source of nuclear factor-kappaB and c-Jun N-terminal kinase mediated inflammation in patients with coronary artery disease. *J. Clin. Endocrinol. Metab.* 94 261–267. 10.1210/jc.2007-2579 18984670

[B4] Baker A. R. Silva N. F. Quinn D. W. Harte A. L. Pagano D. Bonser R. S. (2006). Human epicardial adipose tissue expresses a pathogenic profile of adipocytokines in patients with cardiovascular disease. *Cardiovasc. Diabetol.* 5:1. 16412224 10.1186/1475-2840-5-1 PMC1352345

[B5] Cacciatore F. Abete P. Mazzella F. Furgi G. Nicolino A. Longobardi G. (2012). Six-minute walking test but not ejection fraction predicts mortality in elderly patients undergoing cardiac rehabilitation following coronary artery bypass grafting. *Eur. J. Prev. Cardiol.* 19 1401–1409. 10.1177/1741826711422991 21933832

[B6] Chaldakov G. N. Stankulov I. S. Aloe L. (2001). Subepicardial adipose tissue in human coronary atherosclerosis: another neglected phenomenon. *Atherosclerosis* 154 237–238. 10.1016/s0021-9150(00)00676-6 11190652

[B7] Chen P. S. Turker I. (2012). Epicardial adipose tissue and neural mechanisms of atrial fibrillation. *Circ. Arrhythm. Electrophysiol.* 5 618–620. 10.1161/circep.112.974956 22895598 PMC3438902

[B8] Eroglu S. Sade L. E. Yildirir A. Bal U. Ozbicer S. Ozgul A. S. (2009). Epicardial adipose tissue thickness by echocardiography is a marker for the presence and severity of coronary artery disease. *Nutr. Metab. Cardiovasc. Dis.* 19 211–217. 10.1016/j.numecd.2008.05.002 18718744

[B9] Ferrara N. Abete P. Corbi G. Paolisso G. Longobardi G. Calabrese C. (2005). Insulin-induced changes in beta-adrenergic response: an experimental study in the isolated rat papillary muscle. *Am. J. Hypertens.* 18 348–353. 10.1016/j.amjhyper.2004.10.006 15797652

[B10] Fontes-Carvalho R. Fontes-Oliveira M. Sampaio F. Mancio J. Bettencourt N. Teixeira M. (2014). Influence of epicardial and visceral fat on left ventricular diastolic and systolic functions in patients after myocardial infarction. *Am. J. Cardiol.* 114 1663–1669. 10.1016/j.amjcard.2014.08.037 25306552

[B11] González A. Schelbert E. B. Díez J. Butler J. (2018). Myocardial interstitial fibrosis in heart failure:biological and translational perspectives. *J. Am. Coll. Cardiol.* 71 1696–1706. 10.1016/j.jacc.2018.02.021 29650126

[B12] Gulati A. Jabbour A. Ismail T. F. Guha K. Khwaja J. Raza S. (2013). Association of fibrosis with mortality and sudden cardiac death in patients with non ischemic dilated cardiomyopathy. *JAMA* 309 896–908. 23462786 10.1001/jama.2013.1363

[B13] Hafstad A. D. Nabeebaccus A. A. Shah A. M. (2013). Novel aspects of ROS signalling in heart failure. *Basic Res. Cardiol.* 108 359. 10.1007/s00395-013-0359-8 23740217

[B14] Heymans S. Hirsch E. Anker S. D. Aukrust P. Balligand J. L. Cohen-Tervaert J. W. (2009). Inflammation as a therapeutic target in heart failure? A scientific statement from the translational research committeeof the heart failure association of the European Society of Cardiology. *Eur. J. Heart. Fail.* 11 119–129. 10.1093/eurjhf/hfn043 19168509 PMC2639409

[B15] Iacobellis G. Corradi D. Sharma A. M. (2005). Epicardial adipose tissue: anatomic, biomolecular and clinical relationships with the heart. *Nat. Clin. Pract. Cardiovasc. Med.* 2 536–543. 10.1038/ncpcardio0319 16186852

[B16] Iacobellis G. Ribaudo M. C. Zappaterreno A. Iannucci C. V. Leonetti F. (2004). Relation between epicardial adipose tissue and left ventricular mass. *Am. J. Cardiol.* 94 1084–1087. 10.1016/j.amjcard.2004.06.075 15476634

[B17] Jacobson A. F. Senior R. Cerqueira M. D. Wong N. D. Thomas G. S. Lopez V. A. (2010). Myocardial iodine-123 meta-iodobenzylguanidine imaging and cardiac events in heart failure. Results of the prospective ADMIRE-HF (AdreView Myocardial Imaging for Risk Evaluation in Heart Failure) study. *J. Am. Coll. Cardiol.* 55 2212–2221. 10.1016/j.jacc.2010.01.014 20188504

[B18] Jiang Y. H. Jiang P. Yang J. L. Ma D. F. Lin H. Q. Su W. G. (2015). Cardiac dysregulation and myocardial injury in a 6-hydroxydopamine-induced rat model of sympathetic denervation. *PLoS One* 10:e0133971. 10.1371/journal.pone.0133971 26230083 PMC4521861

[B19] Liguori I. Russo G. Curcio F. Bulli G. Aran L. Della-Morte D. (2018). Oxidative stress, aging, and diseases. *Clin. Interv. Aging* 13 757–772. 29731617 10.2147/CIA.S158513 PMC5927356

[B20] Lin Y. K. Chen Y. C. Chen J. H. Chen S. A. Chen Y. J. (2012). Adipocytes modulate the electrophysiology of atrial myocytes: implications in obesity-induced atrial fibrillation. *Basic Res. Cardiol.* 107:293. 10.1007/s00395-012-0293-1 22886089

[B21] Mazurek T. Zhang L. Zalewski A. Mannion J. D. Diehl J. T. Arafat H. (2003). Human EAT is a source of inflammatory mediators. *Circulation* 108 2460–2466. 14581396 10.1161/01.CIR.0000099542.57313.C5

[B22] Packer M. (2018). Epicardial adipose tissue may mediate deleterious effects of obesity and inflammation on the myocardium. *J. Am. Coll. Cardiol.* 71 2360–2372. 10.1016/j.jacc.2018.03.509 29773163

[B23] Parisi V. Petraglia L. D’Esposito V. Cabaro S. Rengo G. Caruso A. (2019a). Statin therapy modulates thickness and inflammatory profile of human epicardial adipose tissue. *Int. J. Cardiol.* 274 326–330. 10.1016/j.ijcard.2018.06.106 30454723

[B24] Parisi V. Petraglia L. Formisano R. Caruso A. Grimaldi M. G. Bruzzese D. (2019b). Validation of the echocardiographic assessment of epicardial adipose tissue thickness at the Rindfleisch fold for the prediction of coronary artery disease. *Nutr. Metab. Cardiovasc. Dis.* 30 99–105. 10.1016/j.numecd.2019.08.007 31648886

[B25] Parisi V. Rengo G. Pagano G. D’Esposito V. Passaretti F. Caruso A. (2015). Epicardial adipose tissue has an increased thickness and is a source of inflammatory mediators in patients with calcific aortic stenosis. *Int. J. Cardiol.* 186 167–169. 10.1016/j.ijcard.2015.03.201 25819894

[B26] Parisi V. Rengo G. Perrone-Filardi P. Pagano G. Femminella G. D. Paolillo S. (2016). Increased epicardial adipose tissue volume correlates with cardiac sympathetic denervation in patients with heart failure. *Circ. Res.* 118 1244–1253. 10.1161/CIRCRESAHA.115.307765 26926470

[B27] Perazzolo Marra M. De Lazzari M. Zorzi A. Migliore F. Zilio F. Calore C. (2014). Impact of the presence and amount of myocardial fibrosis by cardiac magnetic resonance on arrhythmic outcome and sudden cardiac death in nonischemic dilated cardiomyopathy. *Heart Rhythm* 11 856–863. 10.1016/j.hrthm.2014.01.014 24440822

[B28] Ponikowski P. Voors A. A. Anker S. D. Bueno H. Cleland J. G. F. Coats A. J. S. (2016). 2016 ESC Guidelines for the diagnosis and treatment of acute and chronic heart failure: the task force for the diagnosis and treatment of acute and chronic heart failure of the European Society of Cardiology (ESC)Developed with the special contribution of the Heart Failure Association (HFA) of the ESC. *Eur. Heart J.* 37 2129–2200. 27206819 10.1093/eurheartj/ehw128

[B29] Salgado-Somoza A. Teijeira-Fernández E. Fernández A. L. González-Juanatey J. R. Eiras S. (2010). Proteomic analysis of epicardial and subcutaneous adipose tissue reveals differences in proteins involved in oxidative stress. *Am. J. Physiol. Heart Circ. Physiol.* 299 H202–H209. 10.1152/ajpheart.00120.2010 20435850

[B30] Schelbert E. B. Piehler K. M. Zareba K. M. Moon J. C. Ugander M. Messroghli D. R. (2015). Myocardial fibrosis quantified by extracellular volume is associated with subsequent hospitalization for heart failure, death, or both across the spectrum of ejection fraction and heart failure stage. *J. Am. Heart Assoc.* 4:e002613. 10.1161/JAHA.115.002613 26683218 PMC4845263

[B31] Takano T. Tanaka K. Ozaki K. Sato A. Iijima K. Yanagawa T. (2018). Clinical predictors of recurrent ventricular arrhythmias in secondary prevention implantable cardioverter defibrillator recipients with coronary artery disease - lower left ventricular ejection fraction and incomplete revascularization. *Circ. J.* 24 82 3037–3043. 10.1253/circj.CJ-18-0646 30305485

[B32] Van der Bijl P. Delgado V. Bax J. J. (2017). Sudden cardiac death: the role of imaging. *Int. J. Cardiol.* 237 15–18. 28284506 10.1016/j.ijcard.2017.03.010

[B33] Venteclef N. Guglielmi V. Balse E. Gaborit B. Cotillard A. Atassi F. (2015). Human epicardial adipose tissue induces fibrosis of the atrial myocardium through the secretion of adipo-fibrokines. *Eur. Heart J* 36 795–805. 10.1093/eurheartj/eht099 23525094

[B34] Wagner K. B. Felix S. B. Riad A. (2014). Innate immune receptors in heart failure: side effect or potential therapeutic target? *World J. Cardiol.* 6 791–801. 10.4330/wjc.v6.i8.791 25228958 PMC4163708

[B35] Zhou Q. Zhang L. Wang K. Xu X. Ji M. Zhang F. (2014). Effect of interconnection between cervical vagus trunk, epicardial fat pad on sinus node function, and atrial fibrillation. *Pacing. Clin. Electrophysiol.* 37 356–363. 10.1111/pace.12265 24111726

